# Book Review

**DOI:** 10.1120/jacmp.v4i4.2509

**Published:** 2003-09-01

**Authors:** Nzhde Agazaryan

**Affiliations:** ^1^ UCLA School of Medicine 200 UCLA Medical Plaza, Suite B265 Los Angeles CA 90095

## Abstract

**Proceedings of the 2003 AAPM Summer School, Intensity Modulated Radiation Therapy‐The State Of The Art.** Colorado Springs, CO, 22–26 June 2003, edited by Jatinder R. Palta, Ph.D and T. Rockwell Mackie, Ph.D., Medical Physics Publishing, Madison, WI. Price: $100 (AAPM Members $80). ISBN: 1‐30524‐16‐1.

PACS number(s): 87.53.Bn, 87.53.Kn

This book is edited by two recognized and experienced medical physicists and includes well written and carefully edited lectures presented by 72 contributing experts in the field of medical physics. The editors themselves contributed to several chapters of the book and have organized the contributions in a coherent manner, starting with an elegant historical perspective on intensity modulated radiation therapy (IMRT) by Steve Webb, and concluding with chapters about the future of IMRT and novel uses of IMRT by Battista *et al*. and Welsh *et al*., respectively.

The content of the book reflects the goal of the program of presenting the state of current IMRT planning and delivery technology, issues associated with a clinical implementation of IMRT, and forecasting the role of IMRT in the future of radiation oncology. Although some of the detailed descriptions of optimization algorithms may be challenging to follow, the overall level of presentation is suitable for practicing medical physicists who are not experts in IMRT.

The book offers a wealth of information on IMRT that otherwise would require extensive research to acquire. However, some chapters in the book include sections that have been published elsewhere in nearly identical format. Additionally, recommendations of the Intensity Modulated Radiation Therapy Collaborative Working Group are not followed in terms of IMRT nomenclature by some of the authors. For example, it is the recommendation of this group that the term segmental should replace terms step‐and‐shoot, stop‐and‐shoot, move‐and‐shoot, multiple segment, multi‐segment, and other similar permutations used to describe segmental multileaf collimation (SMLC).

In conclusion, as with many other AAPM Summer School proceedings, presented chapters offer comprehensive, up‐to‐date, and practical information. The book is recommended as a worthwhile addition to the personal collection of books for anyone interested in therapeutic medical physics, and should be in most radiation oncology department libraries.

